# Pathologic Rupture of the Spleen in Mantle-Cell-Type Non-Hodgkin's Lymphoma

**DOI:** 10.1155/2012/351275

**Published:** 2012-04-22

**Authors:** Christopher B. Tan, Dhyan Rajan, Sumreen Majeed, Shadab Ahmed, Lester Freedman, Paul Mustacchia

**Affiliations:** ^1^Department of Internal Medicine, Nassau University Medical Center, East Meadow, NY 11554, USA; ^2^Department of Infectious Disease, Nassau University Medical Center, East Meadow, NY 11554, USA; ^3^Department of Pathology, Nassau University Medical Center, East Meadow, NY 11554, USA; ^4^Department of Gastroenterology, Nassau University Medical Center, East Meadow, NY 11554, USA

## Abstract

Mantle cell lymphoma (MCL) accounts for less than 10 percent of all non-Hodgkin's lymphoma (NHL). Pathologic or spontaneous rupture of the spleen has been reported in patients with lymphoma; however only 5 cases have been reported in patients with MCL. Although splenomegaly occurs frequently in patients with MCL, spontaneous splenic rupture is rare. We present a case of a 51-year-old female with MCL, who presented to the medical emergency room with splenic rupture. This case illustrates that clinicians should be aware of the incidence and presentation of patients with MCL and spontaneous splenic rupture, as early detection and heightened suspicion may prevent potentially fatal outcomes.

## 1. Introduction

Pathologic or spontaneous rupture of the spleen is a rare occurrence and is usually associated with infections or malignancy [1]. Although spontaneous splenic ruptures from an infectious etiology such as malaria, infectious mononucleosis, and typhoid have been reported, splenic rupture secondary to malignancy is infrequent [2]. Spontaneous splenic rupture in patients with hematologic malignancies was first described by Rokitansky et al. in [3], followed by further reports of spontaneous splenic rupture in patients with leukemia over the next century. In 1966, Knoblich et al. [4] reported three cases of spontaneous splenic rupture in patients with leukemia [1]; since 1966, approximately 146 more cases of splenic rupture in the setting of malignancy have been reported. Although the vast majority of these cases occurred in patients with hematologic malignancies such as leukemia, approximately 25 percent of these cases occurred in patients with non-Hodgkin's lymphoma (NHL). Mantle cell lymphoma (MCL), which accounts only for 3 to 10 percent of all NHL, has been rarely associated with splenic rupture, with only five cases being reported in our literature review [5–9]. We present a case of a spontaneous splenic rupture in a 51-year-old female with MCL.

## 2. Case Presentation

A 51-year-old African American female with a medical history significant for diabetes mellitus and NHL presented to the medical emergency room with complaints of dizziness for nearly 12 hours. The patient stated that she noticed the abrupt onset of dizziness while at rest, which she described as persistent and not related to changes in position. She denied any nausea, vomiting, headaches, fevers, chills, abdominal pain, and fatigue. She denied the use of any medications at home as her diabetes mellitus was diet controlled. She was recently diagnosed with NHL 2 months before, and was currently being evaluated by her oncologist for varying therapeutic options. She denied the use of any illicit drugs, tobacco, and alcohol. Family history was noncontributory, including the absence of any malignancy.

In the emergency room, the patient was lethargic, pale, and appeared to be in moderate distress. Vital signs recorded on initial examination revealed the presence of hypotension and tachycardia, with fluid resuscitation only modestly increasing the patient's systolic blood pressure to 80 mm Hg. Physical examination was remarkable for moderate abdominal distention with marked hepatosplenomegaly. There was no abdominal tenderness noted.

Complete blood count (CBC) was significant for hemoglobin of 6.1 g/dL, hematocrit of 19 percent, and a platelet count of 41,000/mm^3^. An emergent chest radiograph revealed the presence of an elevated right diaphragm, likely secondary to marked hepatosplenomegaly. Prior to the initiation of further diagnostic and therapeutic interventions, the patient was again noted to be hypotensive with a systolic blood pressure of 60 mm Hg. The patient was now minimally responsive with absent peripheral pulses, thus cardiopulmonary resuscitation was initiated. Despite numerous therapeutic interventions such as the administration of vasopressors, mechanical intubation, and other measures outlined in Advanced Cardiac Life Saving (ACLS) protocols, the patient expired.

**Figure 1:**
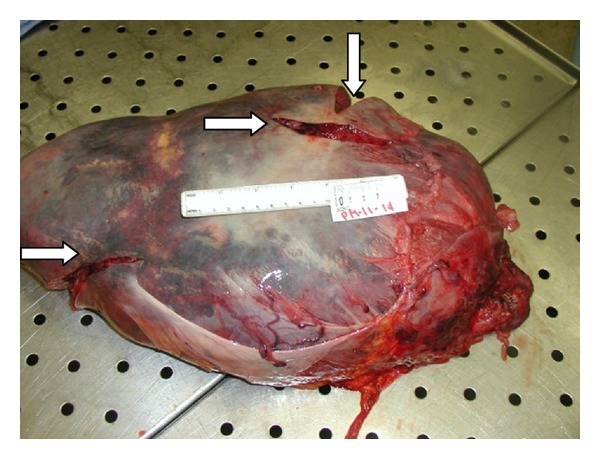
Gross specimen of a massive spleen weighing 6400 grams Note the multiple capsular lacerations present (arrows).

**Figure 2:**
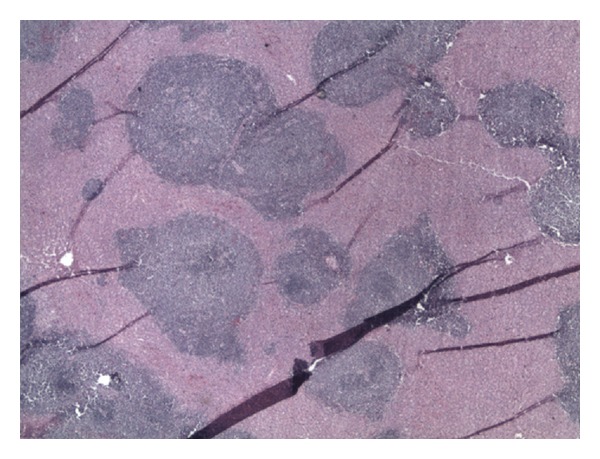
Liver tissue with a nodular pattern of lymphocytic infiltration noted.

**Figure 3:**
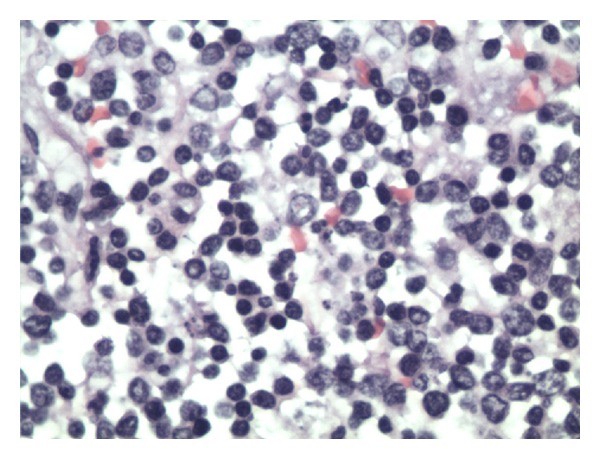
Histopathology of splenic tissue. Note the presence of small cleaved cells (centrocytes) suggestive of mantle cell non-Hodgkin's lymphoma.

**Figure 4:**
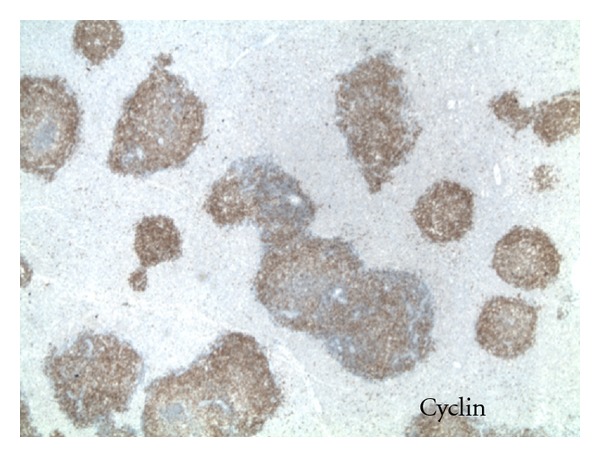
Immunohistochemical staining positive for cyclin D-1 correlating with the t(11; 13) translocation of the short arm of chromosome 13, which is highly specific for MCL.

**Figure 5:**
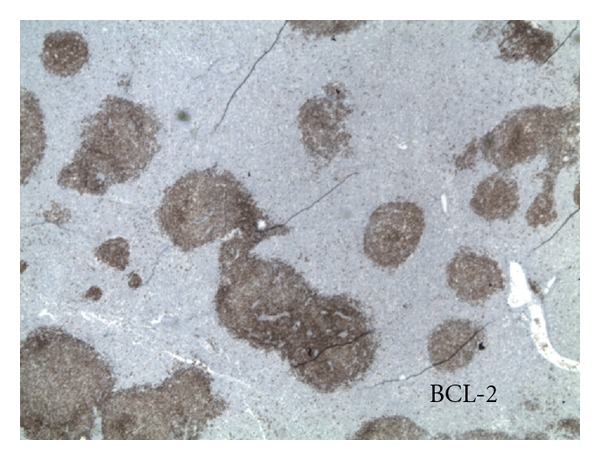
Immunohistochemical staining revealing BCL-2 positivity.

An autopsy performed revealed a markedly enlarged spleen measuring 30 cm × 20 cm × 10 cm, weighing 6400 grams, with multiple foci of capsular lacerations noted (Figure 1). Hemoperitoneum of fresh and clotted blood amounting to approximately 1000 mL was also noted. Histopathologic examination of splenic tissue showed massive nodular infiltration of the spleen by small cleaved lymphocytes (Figures 2 and 3). Immunohistochemistry results supported the diagnosis of NHL of mantle cell type (Figures 4 and 5; Table 1).

## 3. Discussion

MCL accounts for less than 10 percent of all NHL. Typical morphologic features of MCL are monomorphic lymphoid proliferation of small- to medium-sized cells with slight to markedly irregular nuclear contours [5, 10]. The diagnosis of MCL is usually confirmed by immunohistochemical staining, with suggestive findings including CD5, CD20, and most importantly cyclin D1 positivity. Overexpression of cyclin D1 correlates with the t(11; 13) translocation of the short arm of chromosome 13 which is highly specific for MCL and is used to differentiate with other potential morphologic mimics such as chronic lymphocytic leukemia/small cell lymphoma (CLL), follicular lymphoma (FL), and marginal zone lymphoma (MZL) [6].

Splenic enlargement occurs in nearly 40 percent of patients with MCL; however splenic rupture is infrequent. The suggested mechanism of splenic rupture in malignancy includes splenic enlargement, splenic and capsular infiltration by lymphoma, and eventual splenic infarction leading to capsular hemorrhage [1, 5, 11, 12]. Although splenic enlargement itself poses the greatest risk for potential splenic rupture, other factors have been postulated to account for such occurrences [1, 11]. The risk of splenic rupture increases dramatically with age. This observation is most likely related to the difference in hematologic cell types and anatomical abnormalities rendering the spleen more vulnerable [11, 12]. Induction chemotherapy directed towards the preexisting malignancy may also be a potential risk factor for splenic rupture. It is believed that the release of enzymatic content of the cells shortly after induction chemotherapy can lead to splenic damage that ultimately leads to pathologic rupture [11]. Fungal infections in patients with malignancy can also predispose patients to splenic rupture. Fungal granulomas of the spleen may potentiate rupture by further weakening the capsule in an already compromised spleen. This is especially true with *Aspergillus spp.* which are angioinvasive organisms causing erosion and occlusion of blood vessels, thereby increasing the risk for splenic rupture [12]. Variation in specific subtypes of MCL, male sex, and aggressiveness of disease have also been thought to be additional risks for splenic rupture in patients with MCL [1, 8, 11–13]. Special attention to these risk factors with prophylactic splenectomy in patients with multiple risks could prevent these fatal outcomes.

The presentation of splenic rupture in a patient varies, often posing a diagnostic challenge for clinicians. The most common clinical feature of splenic rupture is abdominal pain, which has been reported to be present in nearly 70 percent of cases. The absence of abdominal pain; however, should not waver the clinical suspicion of splenic rupture in patients with malignancy and potential risk factors for this occurrence. In a study by Görg et al. [14], 41 cases of patients with splenic rupture were reviewed, with only 60 percent of them ever complaining of abdominal pain prior to diagnosis [14]. Griffiths et al. report a case of splenic rupture and subsequent hemoperitoneum in the absence of abdominal pain, similar to that of our described case [15]. Nausea, vomiting, fever, and chills have been reported in cases of splenic rupture, as have signs of hemodynamic compromise such as hypotension and tachycardia. Kehr's sign, which is defined as left hypochondriac pain radiating to the left shoulder, occurs in nearly 20 percent of patients, however, is neither sensitive nor specific for splenic rupture [11].

Diagnosis of a spontaneously ruptured spleen relies on both clinical and confirmatory imaging studies [16]. The sensitivity of ultrasound for detecting splenic pathology ranges from 72 to 78% with a specificity of 91–100% [14]. Combined with the availability, portability, and minimal patient preparation, ultrasound is a vital tool in screening for a ruptured spleen. Other diagnostic tests include computed tomography (CT), positron emission tomography (PET), and diagnostic peritoneal lavage [11, 15]. Prompt diagnosis and surgical intervention for a ruptured spleen are essential for patient survival since the mortality rate from splenic rupture approaches 100% [1, 15, 16]. Given the high mortality rate of splenic rupture, heightened suspicion for such an occurrence is warranted.

In our literature review, no single laboratory or imaging studies will accurately predict impending splenic rupture. The earliest evidence of splenic injury would include low-grade lesions such as perisplenic blood without subcapsular splenic hematoma or intraparenchymal bleed referred to as a “sentinel clot” [14]. This low-grade splenic injury has the potential to develop into a subcapsular hematoma with subsequent splenic rupture [14, 15]. Combined with lymphoma-induced thrombocytopenia and coagulopathy, these low-grade lesions are at higher risk for progression in lymphoma than in other causes of splenomegaly. Athale et al. suggested that improved imaging techniques and increased utilization of imaging studies may account for a rise in incidental detection of “preclinical” splenic rupture. Surveillance imaging using ultrasound or CT scanning for the presence of a “sentinel clot” in patients with hematologic malignancies is therefore recommended [12].

The understanding of potential splenic rupture should guide clinicians in regards to therapeutic options for patients with MCL. Treatment of MCL depends on the stage of the disease and the presence of B symptoms. Early stages I and II, which accounts for 15 to 25% of low-grade lymphomas, radiotherapy with or without combination chemotherapy represents the treatment of choice and may be applied with curative intent. Asymptomatic advanced MCL, stages III and IV, may be observed carefully until the disease progresses and/or symptoms occur since a fraction of patients present with a rather indolent form of MCL. Immediate treatment is advocated in advanced symptomatic patients or highly aggressive lymphoma who can tolerate combined chemotherapy. Caution during induction chemotherapy is suggested, as this is known to be a potential risk for worsening cytopenia, tumor lysis syndrome, and splenic rupture [12, 17].

In the event of spontaneous splenic rupture, both nonsurgical and surgical modalities exist. Although nonoperative management including packing and arterial embolization has been attempted, nonoperative management has been associated with a high mortality and is seldom used today [1, 8].

Splenectomy in NHL plays an important role in palliation of symptoms of splenomegaly such as left upper quadrant pain, early satiety, weakness, and fatigue. Other indications for splenectomy include cytopenia, residual splenomegaly in patients successfully responding to chemotherapy in other sites, and a rapidly enlarging spleen at risk for rupture prior to enrollment in clinical drug trials [18–21]. Although splenectomy is often performed to alleviate symptoms of splenomegaly, clinicians should be aware of potential splenic rupture in patients with NHL, including MCL. This awareness, along with the identification of risk factors for spontaneous splenic rupture, may prompt prophylactic splenectomy in appropriate patients. Prophylactic splenectomy may therefore aid in preventing the possibly fatal consequence of splenic rupture in patients with MCL. 

## Figures and Tables

**Table 1:** Immunohistochemical staining results.

Antigen	Result
CD 20	Positive
CD 10	Negative
CD 5	Positive
BCL-2	Positive
CYCLIN D-1	Positive
CD 23	Negative

CD: cluster of differentiation; BCL: B-cell lymphoma.
